# A randomized, crossover, placebo controlled, double-blind trial of the effects of tiotropium-olodaterol on neuromuscular performance during exercise in COPD

**DOI:** 10.1152/japplphysiol.00332.2021

**Published:** 2022-03-24

**Authors:** Min Cao, Robert A. Calmelat, Peter Kierstead, Nicolo Carraro, William W. Stringer, Janos Porszasz, Richard Casaburi, Harry B. Rossiter

**Affiliations:** ^1^Rehabilitation Clinical Trials Center, The Lundquist Institute for Biomedical Innovation at Harbor-UCLA Medical Center, Torrance, California; ^2^Department of Respiratory and Critical Care Medicine, Beijing Chest Hospital, Capital Medical University, Beijing, China; ^3^Antioch Medical Center, Pulmonary Medicine, Antioch, California; ^4^High Specialization Rehabilitation Hospital, ORAS, Motta di Livenza, Italy

**Keywords:** bronchodilation, dynamic hyperinflation, exercise intolerance, fatigue, isokinetic dynamometry

## Abstract

Exercise intolerance in chronic obstructive pulmonary disease (COPD) is associated with dyspnea, reduced inspiratory capacity (IC) and occurs with a neuromuscular “power reserve,” i.e., an acute ability to increase isokinetic locomotor power. This power reserve is associated with resting forced expiratory volume in 1 s (FEV_1_)/forced vital capacity (FVC) suggesting that treatments to target pulmonary function may protect neuromuscular performance and extend whole body exercise in COPD. We, therefore, tested whether combination long-acting β-agonist and muscarinic antagonist bronchodilator therapy [long-acting muscarinic antagonist (LAMA) + long-acting β-agonist (LABA); Stiolto Respimat] would ameliorate the decline in neuromuscular performance and increase endurance time during constant power cycling at 80% peak incremental power. Fourteen patients with COPD (4 female; 64 [58, 72] yr; FEV_1_ 67% [56%, 75%] predicted; median [25th, 75th percentile]) participated in a randomized, placebo-controlled crossover trial (NCT02845752). Pulmonary function and cardiopulmonary exercise responses were assessed before and after 1 wk of treatment, with 2 wk washout between conditions. Performance fatigue was assessed using an ∼4-s maximal isokinetic cycling effort at preexercise, isotime, and intolerance. Isotime was the shorter exercise duration of the two treatment conditions. Significance was assessed using ANOVA with treatment as fixed factor and subject as random factor. FEV_1_ was greater with LAMA + LABA versus placebo (1.81 [1.58, 1.98] L vs. 1.72 [1.29, 1.99] L; *P* = 0.006), but IC at isotime, performance fatigue at isotime, and constant power endurance time were not different between conditions (each *P* > 0.05). A modest (∼95 mL) increase in FEV_1_ following 1 wk of combination LAMA + LABA treatment did not alleviate neuromuscular performance fatigue or enhance cycle exercise tolerance in patients with mild-to-severe COPD with largely preserved “static” lung volumes.

**NEW & NOTEWORTHY** Bronchodilation is known to increase forced expiratory volume in 1 s (FEV_1_) and reduce hyperinflation in COPD. In a randomized controlled trial, we investigated whether combined inhaled long-acting β-agonist and muscarinic antagonist would alleviate maximal voluntary neuromuscular performance fatigue or enhance maximal muscle activation during cycling in patients with COPD. Despite increased FEV_1_, combination bronchodilator therapy did not reduce neuromuscular performance fatigue or enhance muscle activity or exercise tolerance in patients with mild-to-severe COPD.

## Graphical abstract

**Figure:**
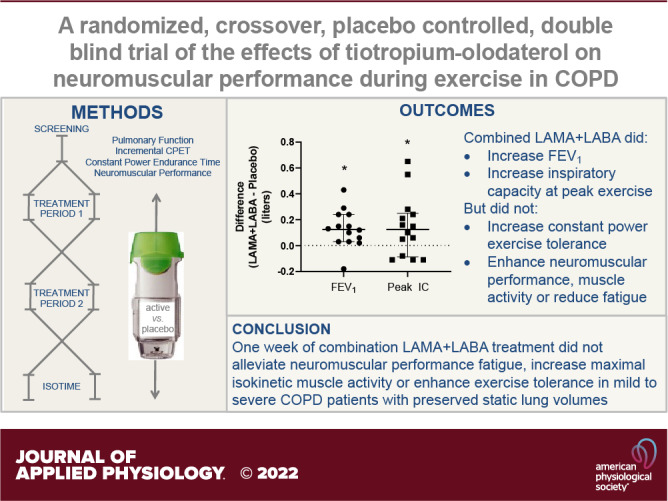

## INTRODUCTION

Chronic obstructive pulmonary disease (COPD) is characterized by dyspnea on exertion and exercise intolerance consequent to expiratory flow limitation, dynamic hyperinflation, gas exchange abnormalities, and skeletal muscle dysfunction (1, 2). As in many disease states, exercise intolerance in patients with COPD is strongly associated with morbidity, mortality, and quality of life (3, 4). The mechanism that limits the ability to maintain large-muscle-mass exercise (i.e., task failure or exercise intolerance), such as walking or cycling in COPD, is therefore of major importance and remains poorly understood.

Patients with COPD have skeletal muscle remodeling that predisposes toward increased muscle fatigue during exercise, where muscle fatigue is defined as the reduction in muscle force and/or shortening velocity that is quickly recoverable with rest (1). Evoked contractions of locomotor muscle following exercise show that patients with COPD have a greater decline in stimulated twitch force for a given absolute or relative work task than age-matched controls (5–7). These findings contribute to the notion that skeletal muscle in COPD is a limiting factor for exercise (8). However, using instantaneous maximal isokinetic efforts, we showed that, despite heightened fatigability, patients with COPD retained a large neuromuscular “power reserve” at peak exercise (9, 10), meaning that muscle fatigue was not a direct limiting factor to sustain whole body exercise in patients with COPD. Instead, we found that the decline in peak isokinetic power (*P*_iso_) during cycling was associated negatively with pulmonary function at rest [forced expiratory volume in 1 s/forced vital capacity (FEV_1_/FVC)] and positively with the magnitude of the ventilatory response at peak exercise [minute ventilation/maximum voluntary ventilation (V̇e/MVV)]. Consistent with this, a recent study using intravenous ascorbate administration, which successfully reduced muscle fatigue during cycling in patients with COPD, did not increase exercise tolerance, corticospinal excitability, or ameliorate exertional dyspnea (11). Together, these findings suggest that some features of pulmonary function contributed strongly to heightened fatigability in patients with COPD, perhaps via spinal or supra-spinal inhibition of locomotor muscle activity. The consequence is a dissociation of the normal relationship between muscle fatigue and limiting symptoms.

The purpose of this study was therefore to determine the magnitude of performance fatigue (the exercise-induced decline in *P*_iso_) and activation fatigue (the fraction of the exercise-induced decline in *P*_iso_ that is explainable by reduced maximal voluntary muscle activation) following once-daily combination bronchodilator treatment compared with placebo. For this we used maximal voluntary isokinetic cycling to: *1*) identify limiting components of neuromuscular performance instantaneously at peak exercise; *2*) assess neuromuscular performance using a contractile velocity close to that of the task; and *3*) overcome the complexities of interpreting change in neuromuscular performance where contractile velocity and torque are allowed to vary (9, 12).

Stiolto Respimat is a combined long-acting β-agonist (LABA; olodaterol hydrochloride) and long-acting muscarinic antagonist (LAMA; tiotropium bromide) that reduces expiratory flow limitation, hyperinflation, and dyspnea and increases exercise tolerance in patients with COPD (13). Given that LAMA + LABA treatment should increase FEV_1_ and inspiratory capacity, we used a randomized, double-blind, placebo-controlled crossover study design to address the hypotheses that, compared with placebo, 1 wk of LAMA + LABA treatment in patients mild-to-severe COPD would *1*) reduce performance fatigue and activation fatigue at isotime and thereby *2*) increase exercise tolerance. To address these hypotheses, we used gas exchange, inspiratory capacity and maximal voluntary isokinetic neuromuscular performance measures during constant power cycle ergometry to intolerance.

## METHODS

### Materials and Methods

#### Participants.

Male and female patients with stable, mild-to-severe COPD were recruited. All provided written informed consent before participation. The study was approved by the local institutional review board (21394-01), complied with the latest revisions of the *Declaration of Helsinki*, and was registered at ClinicalTrials.gov (NCT02845752). Inclusion criteria included aged between 45 and 90 yr; ≥10 pack-yr smoking history; free from a significant disease that might influence exercise tolerance, other than COPD; mMRC dyspnea score ≥ 2. Exclusion criteria included documented history of asthma; documented cardiovascular disease or resting ECG abnormality; unstable COPD (treated with oral corticosteroid medication) or a COPD exacerbation within 3 mo; $\text{S}{\text{p}}_{{\text{O}}_{2}}$ <85% during screening incremental exercise test; completion of pulmonary rehabilitation within the 6 wk before the screening visit; or a limitation of exercise performance as a result of factors other than fatigue or exertional dyspnea (e.g., pain from arthritis, angina, or claudication).

#### Study design.

**Figure 1.**
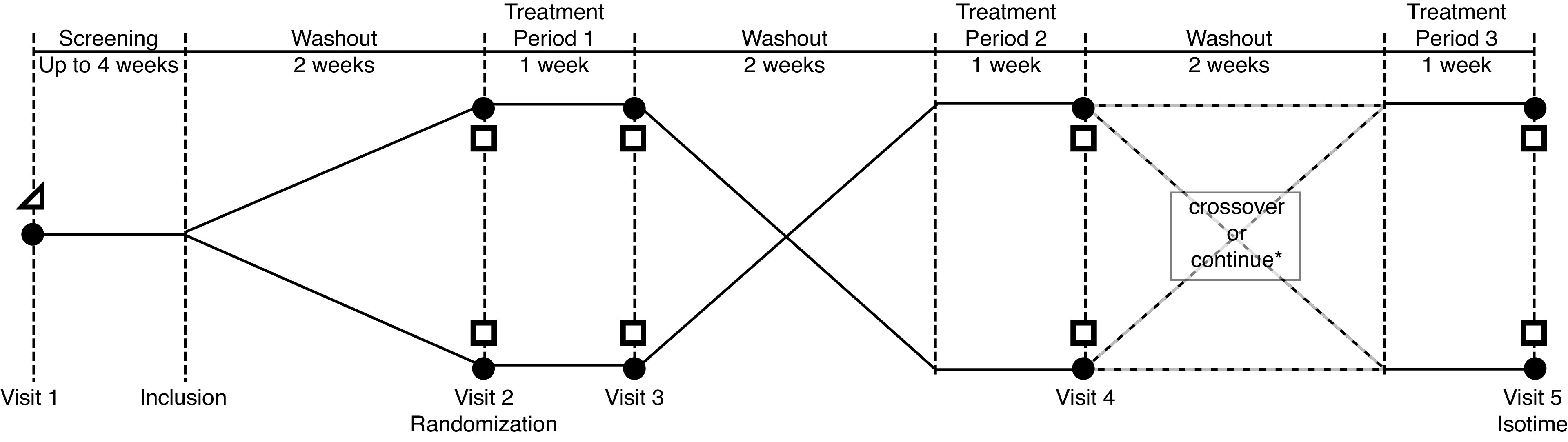
Study design. Assessments included spirometry (filled circle), incremental exercise test (open triangle), and constant power exercise test (open square). All exercise tests were terminated with a maximal voluntary isokinetic cycling power assessment. *Visit 5 (following treatment *period 3*) was conducted using the treatment that resulted in the longest endurance time. Participants either continued on to the same treatment arm as *visit 4*, or crossed-over to the opposite treatment arm after *visit 4*, depending on which treatment resulted in the longer the endurance time at *visits 3* and *4*. At *visit 5*, the constant power exercise test was terminated at isotime with a maximal voluntary isokinetic cycling power assessment. Isotime was the shorter of the two endurance time durations measured at *visits 3* and *4*.

This was a single-center randomized, double-blind, crossover, placebo-controlled trial of Stiolto Respimat, a combined long-acting β-agonist (LABA; olodaterol hydrochloride) and long-acting muscarinic antagonist (LAMA; tiotropium bromide). The study design is illustrated in Fig. 1. Participants were initially screened for eligibility at *visit 1* including pulmonary function and a ramp-incremental exercise test on a cycle ergometer. Eligible participants then discontinued all LAMA and LABA medications for a 2-wk washout period and were provided with a combined short-acting β-agonist and anticholinergic (Combivent Respimat) to use as needed. Participants were instructed not to take any bronchodilator medication on the morning of *visit 2* (randomization/baseline). At *visit 2*, pulmonary function and constant power cycle exercise tests were performed, and participants were randomized to 1 wk of LAMA + LABA or placebo. At the end of each treatment period pulmonary function and exercise assessments were repeated, and followed by another 2-wk washout before crossover. All exercise tests were preceded and terminated by a brief (<5 s) maximal effort voluntary isokinetic cycling task to assess neuromuscular performance. The shorter of the two endurance times at *visits 3* and *4* was taken as isotime. Before *visit 5* (isotime visit), participants again washed out from the preceding treatment for 2 wk and were then given 1 wk of the treatment (LAMA + LABA or placebo) that resulted in the longer endurance time at *visits 3* and *4*. The *visit 5* constant power exercise test was then terminated with a maximal isokinetic effort at isotime; this allowed comparison between treatments of neuromuscular performance and cardiopulmonary variables at isotime.

#### Treatments.

During treatment periods, participants were instructed to take two actuations of a sterile aqueous solution of LAMA + LABA (Stiolto) or placebo, once daily, using the Respimat actuator. The active medication provided 6.2-mg tiotropium bromide monohydrate and 5.5-mg olodaterol hydrochloride per day. The active and placebo medications were packaged in an identical Respimat actuator and provided to the patient in double-blind fashion.

#### Pulmonary function.

Postbronchodilator (albuterol sulfate; ProAir HFA, Teva Respiratory, North Wales, PA) spirometry, body plethysmography [residual volume (RV), functional residual capacity (FRC), total lung capacity (TLC)], and diffusing capacity of the lung for carbon monoxide (D_LCO_) measurements were performed (Vmax Encore with V62J Autobox, CareFusion, San Diego, CA) according to ATS/ERS guidelines (14–16). Maximum voluntary ventilation (MVV) was calculated as 40 times FEV_1_ (15).

#### Ramp-incremental and constant power exercise.

At screening *visit 1*, participants completed a 10 W·min^−1^ ramp-incremental cycle ergometer exercise test to the limit of tolerance (Excalibur Sport PFM, Lode, Groningen, NL). This was used to determine inclusion, characterize exercise capacity, and select power for the constant power exercise tests. At *visits 2–5*, constant power exercise tests were conducted at 80% peak incremental power, with the aim to reach intolerance within 3–8 min (17) (actual range: 3 min 17 s to 6 min 54 s). All exercise tests were preceded by at least 3 min of rest and at least 3 min of unloaded cycling. Intolerance during *visits 1*–*4* was determined as the point at which the participant was unable to maintain at least 50 rpm pedaling cadence, despite strong verbal encouragement. *Visit 5* was terminated at isotime. All tests were followed by at least 3 min of active unloaded cycling while recovery was monitored.

Breath-by-breath gas exchange and ventilation were measured during all exercise tests (VMax Encore, CareFusion, San Diego, CA). The system was calibrated immediately before each testing session. A 3 L syringe (Hans Rudolph Inc., Shawnee, KS) was used to calibrate the mass flow sensor from ∼0.2 to 8.0 L·s^−1^. The CO_2_ and O_2_ analyzers were calibrated using gases of known concentrations (O_2_ 26.0% and 16.0%; CO_2_ 0.0% and 4.0%). Heart rate (HR) was measured from the 12-lead ECG (Cardiosoft, GE Healthcare, Little Chalfont, UK), and oxygen saturation was monitored by pulse oximetry ($\text{S}{\text{p}}_{{\text{O}}_{2}}$; Masimo Corp, Irvine, CA). Ratings of “shortness-of-breath” and “leg effort” using the modified Borg scale (CR-10), and blood pressure (by auscultation), were measured at rest and at 2 min intervals during exercise and recovery. FEV_1_ and inspiratory capacity (IC) were measured in triplicate at rest, and also at 2 min intervals during exercise and recovery. Inspiratory reserve volume (IRV) was calculated from the difference between IC and the spontaneous tidal volume in the 3–4 breaths immediately before the IC measurement.

#### Isokinetic ergometry.

An electromagnetically braked cycle ergometer (Excalibur Sport PFM, Lode BV, Groningen, NL) was instrumented with force transducers in the bottom bracket spindle. Left and right torque (Nm) was measured independently and angular velocity of the crank (rad·s^−1^) was measured every 2° of rotation using three independent sensors sampling in series. During isokinetic efforts, power was calculated every 2° from torque and angular velocity measurements. We have previously shown that there is no systematic difference in the power production between the left and right cranks (9, 10, 12, 18); therefore *P*_iso_ was calculated from power on the right crank averaged over 3 crank revolutions (9, 12) and, where appropriate, reported as 2 times one leg to allow for direct comparison with power output measured at the flywheel.

Before and after each ramp-incremental and constant power test, participants performed the following cycling tasks with pedaling cadence electromagnetically constrained at 70 rpm (isokinetic cycling):

- *Preexercise*: For preexercise isokinetic assessments, participants performed 5 crank-revolution efforts, each at approximately 25%, 50%, 75%, and 100% of maximum effort. Each effort lasted <5 s and was separated by ∼1 min of unloaded cycling. This protocol was repeated once and results used to establish the preexercise slope and intercept of the relationship between muscle activation [by electromyogram (EMG); see below] and isokinetic power (by dynamometry). This also established the preexercise *P*_iso_, which was calculated as the greatest mean power achieved over three consecutive isokinetic crank revolutions appropriately constrained at 70 rpm.
- *End-exercise*: End-exercise isokinetic assessments were made immediately at the limit of tolerance (*visits 1–4*) or at isotime (*visit 5*). For this, the ergometer was switched instantaneously from hyperbolic mode (where the power is constrained constant, but cadence allowed to vary) to isokinetic mode at 70 rpm (where velocity is constrained constant and power is allowed to vary). At the point of switching to isokinetic mode, all resistance from the flywheel was removed, and therefore the pedaling cadence was immediately and rapidly allowed to increase to 70 rpm (constrained by the ergometer). Volunteers were strongly encouraged to give a maximal final effort for 4–5 revolutions (<5 s) before unloaded recovery. This maneuver is similar to the preexercise maximal isokinetic effort, with which the participants were well familiarized and has been validated previously (12).

#### Electromyography.

Surface EMG was measured on the right leg: *vastus lateralis*, *rectus femoris*, *vastus medialis*, *biceps femoris*, and *gastrocnemius lateralis*. The muscle selection reflected the weighted power contributions from knee extension/flexion and plantarflexion (19). Sensor sites were shaved, abraded with gauze, and cleaned. Wireless transmitting silver bipolar parallel-bar surface electrodes (Trigno Wireless System, Delsys Inc., Boston, MA) were placed according to Surface Electromyography for the Non-Invasive Assessment of Muscles (SENIAM) recommendations. During postprocessing, EMG signals were filtered with a second-order Butterworth band-pass filter (3 dB, 10–500 Hz) and smoothed via root mean square (RMS) with a 100 ms window. The peak voltage (μV; from the 100 ms RMS) during each crank revolution was used to quantify of muscle activity. The RMS EMG values from the five muscles of the right leg were averaged to provide an EMG datum to pair with *P*_iso_ produced at the crank from the same leg.

#### Calculation of neuromuscular performance.

The preexercise EMG-*P*_iso_ relationship was characterized as previously described (9, 12). Briefly, RMS EMG values at 25%, 50%, 75%, and 100% effort were normalized to the visit maximum, plotted against the corresponding isokinetic power, and then modeled using least-squares linear regression. Maximal effort EMG and *P*_iso_ measurements at end-exercise were then used to characterize neuromuscular fatigue in the following ways.

The ability to acutely increase isokinetic power output at the limit of tolerance was measured by the power reserve, where:

(*1*) $$\text{Power reserve }\left(\text{W}\right)=(\text{2}\times P_{\text{iso},\text{end-exercise}})-\text{peak incremental power}$$

The absence of a power reserve at V̇o_2peak_ during ramp-incremental exercise represents that neuromuscular performance limits peak-incremental power output.

The tolerance index described the percentage of preexercise peak isokinetic power (*P*_iso_) that was achieved at the limit of tolerance during ramp-incremental exercise (i.e., at V̇o_2peak_):

(*2*) $$\text{Tolerance Index}\left(\%\right)=[\text{peak incremental power}/(\text{2}\times P_{\text{iso},\text{preexercise}})]\times \text{1}00$$

A greater tolerance index represents a greater ability to reach a high percentage of preexercise *P*_iso_ at V̇o_2peak_ during ramp-incremental exercise.

The fatigue index during ramp-incremental exercise described the percentage fall in peak isokinetic power between preexercise and the limit of tolerance (i.e., at V̇o_2peak_):

(*3*) $$\text{Fatigue index}\left(\%\right)=[(P_{\text{iso},\text{pre-exercise}}-P_{\text{iso},\text{end-exercise}})/P_{\text{iso},\text{preexericse}}]\times \text{1}00$$

A greater fatigue index represents a greater relative reduction in *P*_iso_ at V̇o_2peak_ during ramp-incremental exercise.

During constant power exercise, performance fatigue (PF) describes the reduction (in watts) of isokinetic power during a maximum effort as a result of exercise and was calculated as the difference in *P*_iso_ between preexercise (fatigue-free) and end-exercise (intolerance or isotime):

(*4*) $$\text{Performance fatigue}\left(\text{PF},\;\text{W}\right)=P_{\text{iso},\text{preexercise}}-P_{\text{iso},\text{end-exercise}}$$

Activation fatigue (AF) is the fraction (in watts) of PF resulting from reduced muscle activity during maximum isokinetic effort and was calculated from the power equivalent of the reduction in RMS EMG activity:

(*5*) $$\text{Activation fatigue}\left(\text{AF},\text{ W}\right)=P_{\text{iso},\text{preexercise}}-\left(a\times {\text{RMS EMG}}_{\text{end-exericse}}+b\right)$$

where *a* and *b* are the slope and intercept, respectively, of the preexercise linear EMG-*P*_iso_ relationship at 70 rpm (calculated for each individual exercise test).

#### Statistical analyses.

Differences in participant characteristics and ramp-incremental exercise test responses between randomization sequence (LAMA + LABA first vs. placebo first) were assessed by Mann−Whitney *U* test. The effects of treatment (LAMA + LABA vs. placebo) on pulmonary function and responses during constant power exercise tests were assessed as difference from baseline using a univariate general linear model ANOVA, with treatment as fixed factor and subject as random factor. All data were Box-Cox transformed before analysis and reported as untransformed median [25th, 75th quartile]. Statistical significance was accepted at *P* < 0.05.

## RESULTS

Sixteen patients with COPD volunteered to participate, but two patients dropped-out before completing the study. None of the participants used supplemental oxygen. Data from 14 participants (10 male), aged between 51 and 77 with mild-to-severe COPD are reported (Table 1). It is of note that, based on Global Lung Function Initiative (GLI) predictors, only four participants showed evidence of gas trapping (e.g., RV > upper limit of normal) and none showed evidence of hyperinflation (e.g., TLC > upper limit of normal). The seven participants who received placebo first had significantly greater weight (*P* = 0.005) and body mass index (BMI; *P* = 0.004), otherwise demographics, pulmonary function, and ramp-incremental exercise responses were not different between treatment groups at randomization (Tables 1 and 2).

**Table 1.** Baseline participant characteristics

		Randomization Sequence		
	All Participants	LAMA + LABA First	Placebo First	*P* Value
*n* (M/F)	14 (10/4)	7 (5/2)	7 (5/2)	1.000
Race, W/AA	7/7	3/4	4/3	0.593
Age, yr	64 [58, 72]	63 [58, 64]	71 [53, 73]	0.370
Height, cm	171 [167, 177]	170 [168, 174]	175 [163, 179]	0.224
Weight, kg	81 [69, 95]	69 [66, 77]	93 [84, 100]	0.005*
BMI, kg/m^2^	28.2 [24.1, 31.6]	24.2 [22.1, 26.6]	31.6 [31.2, 34.6]	0.004*
Resting $\text{S}{\text{p}}_{{\text{O}}_{2}}$, %	99 [98, 100]	99 [99, 100]	98 [98, 100]	0.176
FEV_1_, L	1.77 [1.46, 2.09]	1.79 [1.37, 2.32]	1.75 [1.49, 2.01]	0.848
FEV_1_, %predicted	66.5 [56.0, 75.3]	69.0 [59.0, 75.0]	66.0 [47.0, 76.0]	0.847
FEV_1_/FVC, %	55.0 [46.5, 58.0]	56.0 [45.0, 58.0]	55.0 [49.0, 58.0]	0.898
GOLD class, 1/2/3	1/10/3	0/6/1	1/4/2	0.420
RV, L	2.36 [1.81, 2.79]	2.30 [1.66, 2.99]	2.40 [1.85, 2.83]	0.886
RV, %predicted	98 [89, 133]	98 [92, 135]	99 [84, 127]	0.721
FRC, L	2.96 [2.51, 3.77]	3.56 [2.39, 3.83]	2.87 [2.63, 3.65]	0.886
FRC, %predicted	105 [83, 124]	107 [80, 135]	99 [84, 111]	0.886
TLC, L	5.71 [4.91, 6.94]	5.97 [4.38, 6.85]	5.50 [4.92, 7.05]	0.668
TLC, %predicted	97 [84, 108]	100 [82, 112]	93 [85, 104]	0.567
RV/TLC	0.43 [0.32, 0.50]	0.43 [0.31, 0.50]	0.44 [0.31, 0.51]	0.668
DL_CO_, mL·min^−1^·mmHg^−1^	13.5 [8.95, 18.6]	11.3 [8, 16.2]	15.2 [9.63, 21.0]	0.391
DL_CO_, %predicted	56 [34, 82]	43 [34, 77]	66 [37, 87]	0.616
IC, L	2.44 [2.15, 2.95]	2.41 [2.14, 2.83]	2.58 [2.10, 3.22]	0.668
IC, %predicted	98 [81, 110]	98 [83, 110]	98 [79, 110]	0.830

Values are median [25th, 75th quartile]. *P < 0.05. BMI, body mass index; DL_CO_, diffusing capacity of the lung for carbon monoxide; FEV_1_, forced expiratory volume in 1 s; FRC, functional residual capacity; FVC, forced vital capacity; GOLD, global initiative on obstructive lung disease; IC, inspiratory capacity; LAMA + LABA, long-acting β-agonist and muscarinic antagonist bronchodilator therapy; M/F, male/female; *n*, number of subjects; RV, residual volume; $\text{S}{\text{p}}_{{\text{O}}_{2}}$, arterial oxygen saturation by pulse oximetry; TLC, total lung capacity; W/AA, White/African American.

**Table 2.** Ramp-incremental cycle ergometry exercise responses

		Randomization Sequence		
	All Participants	LAMA + LABA First	Placebo First	*P* Value
Peak incremental power, W	79 [70, 99]	79 [63, 98]	88 [77, 101]	0.250
Power reserve, W	88 [62, 120]	88 [78, 98]	88 [56, 123]	0.818
Tolerance index, %	24.7 [22.6, 28.2]	24.0 [22.9, 28.9]	24.7 [22.6, 28.2]	0.775
Fatigue index, %	45.4 [32.3, 59.4]	43.4 [32.7, 58.3]	48.5 [29.8, 63.5]	0.749
V̇o_2peak_, L·min^−1^	1.19 [1.01, 1.46]	1.10 [0.87, 1.34]	1.29 [1.10, 1.47]	0.225
V̇o_2peak_, mL·min^−1^·kg^−1^	14.0 [12.9, 18.0]	15.9 [13.3, 20.6]	13.5 [12.9, 17.1]	0.565
Peak HR, min^−1^	117 [103, 123]	117 [101, 122]	112 [103, 132]	1.000
Peak V̇e, L·min^−1^	48.6 [41.7, 51.2]	48.3 [38.4, 50.7]	48.8 [44.9, 52.6]	0.443
Peak V̇e/MVV, %	70.3 [58.9, 86.8]	67.5 [52.4, 92.5]	73.1 [62.0, 86.7]	0.655
Peak $\text{S}{\text{p}}_{{\text{O}}_{2}}$, %	99 [97, 99]	99 [98, 99]	97 [96, 99]	0.265
Peak IC, L	1.96 [1.66, 2.66]	1.84 [1.67, 2.63]	2.10 [1.64, 2.74]	0.565
Peak IRV, L	0.59 [0.34, 0.95]	0.69 [0.36, 0.88]	0.49 [0.34, 1.03]	0.949

Values are median [25th, 75th quartile]. See methods for definition of intolerance index and fatigue index. HR, heart rate; IC, inspiratory capacity; IRV, inspiratory reserve volume; HR, heart rate; LAMA + LABA, long-acting β-agonist and muscarinic antagonist bronchodilator therapy; MVV, maximal voluntary ventilation (calculated from forced expiratory volume in 1 s × 40); $\text{S}{\text{p}}_{{\text{O}}_{2}}$, arterial oxygen saturation by pulse oximetry; V̇e, expired minute ventilation; V̇o_2peak_, peak pulmonary oxygen uptake; W, watts. Total *n* = 14 subjects (*n* = 7 in each randomization sequence group).

Participants had significantly impaired exercise capacity at the screening ramp-incremental test. V̇o_2peak_ was 14.0 [12.9, 18.0] mL·min^−1^·kg^−1^ (Table 2; equivalent to 63.3% [54.3%, 83.8%] predicted) and participants hyperinflated between rest and peak exercise; the median decline in inspiratory capacity was −0.29 [−0.41, −0.26] L. Ramp-incremental exercise was terminated with a large power reserve (88 [62, 120] W; Table 2). The tolerance index at peak ramp-incremental exercise was only 24.7% [22.6%, 28.2%] (Table 2), which represents ∼50% of the value expected for similar-aged control subjects (9, 12). Despite the low peak ramp-incremental work rate (79 [70, 99] W), the fatigue index was 45.4% [32.3%, 59.4%], which represents ∼80% of the value expected for similar-aged control subjects (9, 12). In other words, although patients were not limited during ramp-incremental exercise by neuromuscular performance (a power reserve was present), fatigability was high (the fatigue index was near normal) despite substantially impaired exercise tolerance (the tolerance index was low).

**Figure 2.**
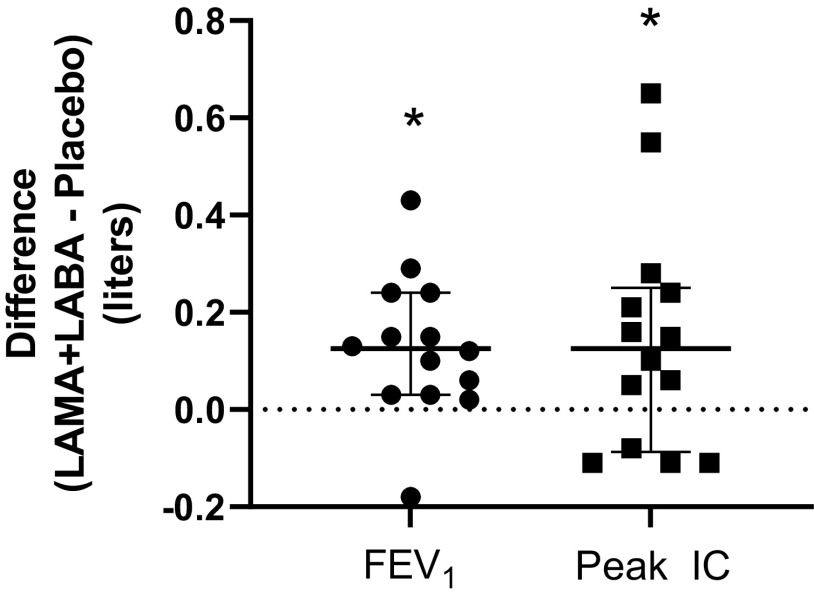
Difference in preexercise (posttreatment) FEV_1_ and inspiratory capacity at peak exercise (Peak IC) between LAMA + LABA versus placebo. Error bars a median and interquartile range. *n* = 14. FEV_1_, forced expiratory volume in 1 s; IC, inspiratory capacity; LAMA + LABA, long-acting β-agonist and muscarinic antagonist bronchodilator therapy.

The effects of LAMA + LABA treatment compared with placebo are shown in Table 3. Patients reported a relatively low burden of symptoms for leg effort and shortness of breath (Table 3). There was a significant effect of LAMA + LABA on FEV_1_ (Fig. 2); both absolute (*P* = 0.007) and % predicted FEV_1_ (*P* = 0.012) were increased (Table 3). However, this did not translate to significant increase in IC (*P* = 0.114) or IRV (*P* = 0.723) at isotime. Despite a significantly greater IC at peak exercise (Fig. 2; *P* = 0.041), the absolute effect size of LAMA + LABA treatment on IC at peak was small (median ∼30 mL between conditions, or 125 [−0.05, 0.23] mL within-subject difference; Fig. 2). Exercise endurance and measures of neuromuscular performance [PF (*P* = 0.190) and AF (*P* = 0.423)] were also not different between treatment conditions.

**Table 3.** Effect of LABA + LAMA versus placebo on pulmonary function, neuromuscular fatigue and endurance time during constant power cycle ergometry in COPD

	LAMA + LABA	Placebo	*P* Value
Preexercise			
Posttreatment FEV_1_, L	1.81 [1.58, 1.98]	1.72 [1.29, 1.99]	0.007*
Posttreatment FEV_1,_ % predicted	71.5 [60.8, 76.0]	67.0 [51.0, 72.0]	0.012*
Isotime			
Isotime leg effort	3 [2, 4]	2.5 [1, 4]	0.162
Isotime shortness of breath	3 [2, 4]	2.5 [1, 4]	0.067
Isotime V̇o_2_, L·min^−1^	1.28 [1.00, 1.46]	1.22 [1.07, 1.45]	0.381
Isotime V̇e, L·min^−1^	50.2 [44.7, 55.5]	45.3 [43.0, 55.4]	0.062
Isotime IC, L	1.94 [1.70, 2.50]	1.90 [1.71, 2.31]	0.114
Isotime IRV, L	0.54 [0.23, 0.90]	0.46 [0.29, 0.85]	0.723
Isotime AF, W	47 [28, 64]	43 [29, 96]	0.774
Isotime PF, W	76 [46, 104]	69 [54, 117]	0.870
Peak exercise			
Endurance time, s	297 [253, 352]	274 [222, 326]	0.759
Peak IC, L	1.91 [1.64, 2.48]	1.88 [1.71, 2.25]	0.041*
Peak IRV, L	0.54 [0.26, 0.86]	0.46 [0.31, 0.60]	0.413
Peak AF, W	58 [33, 74]	50 [27, 80]	0.423
Peak PF, W	75 [53, 103]	77 [61, 117]	0.190

Values are median [25th, 75th quartile]. *P < 0.05. All data were box cox transformed prior to univariate general linear model ANOVA. “Treatment” refers to the treatment conditions (LAMA + LABA or placebo). Leg effort and shortness of breath were assessed using the modified Borg CR-10 scale. Isotime is the shorter exercise duration of the two treatment conditions (LAMA + LABA or placebo). Peak, the time of exercise intolerance. AF, activation fatigue; COPD, chronic obstructive pulmonary disease; FEV_1_, forced expiratory volume in 1 s; IC, inspiratory capacity; IRV, inspiratory reserve volume; LAMA + LABA, long-acting β-agonist and muscarinic antagonist bronchodilator therapy; PF, performance fatigue; V̇e, minute ventilation; V̇o_2_, oxygen uptake. *N* = 7 subjects in each group.

## DISCUSSION

This study tested the hypothesis that combined LAMA + LABA treatment for expiratory flow limitation in patients with mild-to-severe COPD would increase neuromuscular performance during whole body exercise. In a randomized double-blind placebo-controlled trial, we found that combined LAMA + LABA treatment significantly increased FEV_1_ (both absolute and % predicted) and mildly increased inspiratory capacity at peak exercise, but it did not affect neuromuscular performance at isotime or peak exercise. Our participants were characterized by moderate to severe expiratory flow limitation but largely preserved “static” lung volumes. They had reduced aerobic capacity and a large maximal voluntary isokinetic power reserve at peak exercise. Despite this, contrary to our hypothesis, LAMA + LABA treatment did not translate to a reduced performance fatigue or attenuate the decline in maximal voluntary isokinetic locomotor muscle activation, or lead to an increased exercise tolerance.

In a previous study we showed that patients with COPD (9), unlike controls (9, 10, 20), have a large power reserve at intolerance; i.e., they showed an acute ability to increase isokinetic power considerably above the demands of constant power task. This reveals a potential to access better sustained, or increased, power output through an acute relief of the mechanisms that determine intolerance. In the same study, we found in patients with COPD that measures associated with expiratory flow limitation, both at rest (FEV_1_/FVC) and during exercise (peak V̇e/MVV), associated with the decline in maximum voluntary isokinetic power at intolerance during cycle ergometry (9). We therefore hypothesized that an intervention to increase resting FEV_1_ and/or reduce ventilatory demands and/or dynamic hyperinflation during exercise would allow exercise tolerance to be increased in COPD through improved (lesser fatigue of) neuromuscular performance.

The proposed mechanisms limiting whole body exercise are that exercise causes accumulation of peripheral fatigue-associated metabolites (such as intramuscular Pi, H^+^ and interstitial K^+^), and impaired intramuscular calcium handling and sensitivity, which together directly reduce muscle power production to a level below the demands of the exercise task (a direct muscle fatigue limitation to task performance) (21–23) and also that this peripheral metabolite accumulation indirectly limits power production through feedback via group III/IV muscle afferents that activate interneuron inhibition of motor efferent activity in the dorsal horn of spinal cord and/or higher in the neuromuscular chain (indirect central fatigue limitation to performance of exercise) (24). In health, when measured serially during constant power exercise, power reserve declines approximately exponentially, with ∼36% of the total performance fatigue accumulating within the first minute of exercise (25). These dynamics are roughly similar to intramuscular PCr breakdown and Pi accumulation (26, 27), consistent with the concept that the early dynamics of neuromuscular performance fatigue are strongly associated with peripheral muscle metabolite accumulation (25, 28). The later development of central fatigue during time trial exercise suggests that sensory feedback contributes relatively more to limiting muscle activation during sustained exercise tasks than during acute ones (28). This process may contribute to a greater reduction or inhibition in muscle activation in patients with COPD where sensory feedback (e.g., dyspnea) is heightened. This is reflected in our measurement of activation fatigue. However, in our study, LAMA + LABA treatment did not affect dyspnea or pulmonary mechanical variables at isotime; therefore, it is not surprising that activation fatigue also did not differ.

Gagnon et al. showed that inhibition of muscle afferent feedback using intrathecal fentanyl in patients with COPD increased exercise tolerance and allowed them to access a greater level of muscle activity during constant power exercise and generate an increased magnitude of muscle fatigue at intolerance measured using postexercise electrical stimulation (29); this is consistent with our demonstration of a power reserve in COPD (this study and Ref. 9). The effect of intrathecal fentanyl was accompanied by reduced ventilation and ratings of perceived leg fatigue and dyspnea at isotime, again, also consistent with our previous data showing an association between pulmonary function and performance fatigue in patients with COPD (9). Neuromuscular fatigue, measured by potentiated twitch force 10 min after exercise, is also reduced at isotime in patients with COPD using proportional assist ventilation or breathing heliox or hyperoxic gas mixtures (30). An opposing effect of intrathecal fentanyl was observed in healthy subjects—time trial performance was reduced—consequent to impaired O_2_ delivery and earlier accumulation of muscle fatigue limiting performance (24, 31). In relation to this, a previous study of tiotropium + olodaterol on hemodynamic responses to exercise in 20 patients, mostly with moderate and severe COPD, showed that the beneficial effects of treatment on resting and operating lung volumes were not translated into enhanced cardiocirculatory responses (32). Increased exercise tolerance in that study was not mechanistically linked to greater locomotor or respiratory muscle oxygenation, blood flow, and/or O_2_ delivery, suggesting some other mechanism of exercise tolerance benefit. Further, interventions specifically and successfully targeting muscle fatigue in patients with COPD (intravenous ascorbate administration) also did not enhance exercise tolerance (11). This also contributes to the notion that some signal associated with ventilation or dyspnea indirectly limits neuromuscular performance in patients with COPD.

Combined, these previous works and the data from our study suggest that patients with COPD, despite having locomotor muscle remodeling and heightened peripheral fatigability, are not limited in cycling exercise by muscle O_2_ delivery or neuromuscular performance. We speculated that heightened dyspnea in COPD may constitute a sensitizing mechanism, by which increased neuromuscular inhibition and/or reduced motor cortex excitability (through some as yet undefined feedback loop) acts to dissociate the normal relationship between locomotor muscle fatigue and limiting symptoms. Treatment for these symptoms would therefore also alleviate the decline in neuromuscular performance, in a similar fashion to that seen by intrathecal fentanyl administration (29). Clearly, intrathecal fentanyl administration is not intended to be a treatment option for ambulating patients with COPD. For this reason, we sought to determine whether a significant increase in FEV_1_ following LAMA + LABA treatment would alleviate isotime ventilatory demands and/or dynamic hyperinflation and improve neuromuscular performance. However, we saw no effect of LAMA + LABA treatment compared with placebo on our measures of neuromuscular performance.

LAMA + LABA treatment in this patient group led to a significant but modest (∼95 mL) increase in FEV_1_. Is seems likely that this was not sufficient to alleviate ventilatory demands and reduce dynamic hyperinflation, as we did not see a difference in isotime dyspnea, V̇e, IC, or IRV. Although peak IC was significantly greater following LAMA + LABA treatment compared with placebo, this effect was, again, small (∼30 mL). Overall, these effects on pulmonary mechanics appear insufficient to increase exercise tolerance in this small study. This may be related to the fact our patients were predominantly (10/14 patients) moderately severe and that bronchodilator therapy has a greater effect on exercise tolerance in those with more severe obstruction. Although we studied patients with mild-to-severe COPD, we found only ∼5% reversibly of FEV_1_ with 1 wk of combination LAMA + LABA treatment, ∼2% increase in IC at peak exercise, and ∼8% increase (not significant) in exercise endurance time compared with placebo. This reflects only a modest effect of LAMA + LABA treatment in this group. By comparison, in multicenter randomized controlled trials, combined tiotropium + olodaterol had large effects on IC (∼10%) and exercise endurance time (∼19%) after 6 wk of treatment in large cohorts that contained a majority (70%) of moderate [global initiative on obstructive lung disease (GOLD 2)] patients (13). To shorten the overall duration of the study (and participant burden), we elected to study subjects after only 1 wk of treatment, which might have limited the time for which the intervention could act. Despite this, the pharmacokinetics of tiotropium + olodaterol treatment are fast in comparison to the 1-wk treatment duration and is expected to reach peak benefit on FEV_1_ and gas trapping within 24–48 h (33).

In healthy subjects, the size of the power reserve, measured using methods similar to those used in this study, is negatively associated with peripheral fatigue; i.e., a smaller power reserve is correlated with a greater decline in postexercise quadriceps stimulated twitch force (34). This supports the notion that assessment of maximal voluntary isokinetic power at the limit of tolerance provides insight into the degree to which peripheral fatigue developed during the exercise task. The substantial power reserve at V̇o_2peak_ in patients with COPD we found in this study and previously suggests that peripheral fatigue did not develop sufficiently to directly limit exercise tolerance. Of note, a power reserve is also observed in some healthy subjects during constant power exercise (25). This effect is more prevalent during longer (∼20 min) than shorter (∼5–10 min) exercise tasks (35), which may reflect greater contribution of central fatigue during longer tasks in healthy participants. Such a dissociation of the normal relationship between muscle fatigue, which is exacerbated by extended duration, may be heightened in patients with COPD in whom dyspnea is increased compared with controls. If so, we would anticipate greater activation fatigue in patients with COPD, increasing sense of effort and contributing to exercise limitation. Although we did observe a large power reserve, we were unable to alleviate isotime dyspnea or pulmonary mechanical responses to exercise with our LAMA + LABA treatment and therefore activation fatigue in this study did not differ between conditions.

There are several limitations of this study. The sample size is small and the majority of participants were male. The relatively short treatment duration (1 wk) compared with other studies may have contributed to the unexpectedly low response in FEV_1_ and IC. Postrandomization, we measured only spirometric pulmonary function at rest, and therefore were not able to assess potential changes in lung volumes in response to treatment. The a priori sample size was 16–21, for a statistical power (1 – β) of 0.8–0.9, based on an increase in endurance time of 105 s [the proposed minimal clinically important difference (17)]. We therefore do not believe that the final sample size was a major limitation. Rather the primary limitation of the study was that the LAMA + LABA treatment did not have the anticipated effect of increasing exercise tolerance, likely because the average degree of expiratory flow limitation and/or resting hyperinflation were too mild. This means that the hypothesis of whether combination LAMA + LABA therapy increases exercise tolerance in COPD by alleviating performance and/or activation fatigue went untested. An approach that results in a more robust increase in exercise tolerance and reduction in dynamic hyperinflation is required to test this hypothesis more effectively. Only a minority of patients (*n* = 3) were severely obstructed (GOLD 3), which may also have contributed to the modest LAMA + LABA treatment effect in the group as a whole. There was a wide range of DL_CO_ %predicted among participants. However, the association between DL_CO_ %predicted and endurance time increase in the LAMA + LABA condition was not significant (*r*^2^ = 0.25, *P* = 0.194). Nevertheless, the range of DL_CO_ may have confounded the influence of LAMA + LABA in this small study. Finally, peak isokinetic torque is strongly associated with V̇o_2peak_ and exercise tolerance, due to its relation with the volume of muscle mass available for metabolic activity (36). However, there are no established normal values for *P*_iso,preexercise_, and therefore we do not know whether neuromuscular performance in this study was associated with reduced leg muscle mass and/or torque. Future studies of whether LAMA + LABA treatment alleviates performance and/or activation fatigue should focus on patients with more severe expiratory flow limitation and/or resting and exercising hyperinflation with a clear volume response to LAMA + LABA treatment. Additional approaches would be to establish whether performance and/or activation fatigue is affected by alternative treatments known to increase exercise tolerance in COPD, for example, supplemental O_2_, pulmonary rehabilitation, heliox, proportional assist ventilation.

In conclusion, a modest (∼95 mL) FEV_1_ increase in following 1 wk of combination LAMA + LABA treatment did not alleviate neuromuscular performance fatigue, increase maximal isokinetic muscle activity, or enhance cycle ergometer exercise tolerance in this group of patients with mild-to-severe COPD with largely preserved “static” lung volumes.

## GRANTS

This investigation was supported by Boehringer-Ingelheim Investigator-Initiated Study 1237.55. Harry Rossiter is supported by grants from NIH (R01HL151452, P50HD098593, R01DK122767, P2CHD086851) and the Tobacco Related Disease Research Program (T31IP1666). William Stringer has received research grant funding from the Department of Defense, AstraZeneca, Genentech, Roche, and Boehringer Ingelheim involving COPD.

## DISCLOSURES

Harry Rossiter reports consulting fees from Omniox Inc. and is involved in contracted clinical research with GlaxoSmithKline, Novartis, AstraZeneca, Astellas, United Therapeutics, Genentech, and Regeneron. He received grant support from Boehringer-Ingelheim to the institution to conduct this study. William Stringer is a medical director for a pulmonary rehabilitation program and is involved with Data Safety Monitoring Boards that focus on COPD and various biologic therapies. Richard Casaburi discloses consultancy fees from Boehringer-Ingelheim, Regeneron, Genentech, Abbott, and Respinova. He is involved in contracted clinical research with GlaxoSmithKline, Novartis, AstraZeneca, Genentech, and Regeneron. Janos Porszasz and Robert Calmelat are involved in contracted clinical research with AstraZeneca, United Therapeutics, Genentech, and Regeneron. Min Cao, Peter Kierstead, and Nicolo Carraro have nothing to disclose.

## AUTHOR CONTRIBUTIONS

J.P., R.C., and H.B.R. conceived and designed research; M.C., R.A.C., P.K., N.C., W.W.S., J.P., and H.B.R. performed experiments; M.C., N.C., and H.B.R. analyzed data; M.C., N.C., W.W.S., J.P., R.C., and H.B.R. interpreted results of experiments; H.B.R. prepared figures; H.B.R. drafted manuscript; M.C., R.A.C., P.K., N.C., W.W.S., J.P., R.C., and H.B.R. edited and revised manuscript; M.C., R.A.C., P.K., N.C., W.W.S., J.P., R.C., and H.B.R. approved final version of manuscript.
